# From marginalization to transformation: Examining structural injustices and policy impacts on Albania’s Roma and Egyptian communities

**DOI:** 10.12688/openreseurope.23119.1

**Published:** 2026-03-10

**Authors:** Eglantina Dervishi

**Affiliations:** 1 Universiteti i Tiranes Fakulteti i Shkencave Sociale, Tirana, Tirana County, Albania

**Keywords:** conflict-driven transformation, environmental justice, marginalized communities, power dynamics, socioeconomic exclusion, sustainable transitions

## Abstract

This study explores the role of conflict, suffering, and structural injustices in driving transformative processes within Albania’s Roma and Egyptian communities, emphasizing how these experiences challenge consensus-driven approaches to social change. By analyzing systemic inequalities and power struggles through critical theory frameworks, the study highlights the importance of addressing conflict as a catalyst for transformation in the face of global challenges like climate change and the Capitalocene. The research draws on policy reviews and academic literature to examine the intersection of social exclusion, poverty, and environmental justice, revealing the limitations of current ‘just transition’ policies. The findings underscore that conflict and inequality must be acknowledged and addressed to achieve meaningful, justice-oriented transitions, particularly in marginalized communities.

## 1. Introduction

This article aims to contribute to the growing body of scholarship that explores conflict as a key driver of social and environmental transformation. By centering on the experiences of Albania’s Roma and Egyptian communities, it reveals the broader implications of conflict-driven change within the context of climate justice and systemic inequality. Through a critical review of academic literature and policy documents, and by linking local struggles to global challenges, the article underscores the necessity of embedding conflict into governance and policy frameworks as a catalyst for meaningful and lasting transformation.

Transformation processes are often framed within dominant narratives of consensus, harmony and prosperity, emphasizing smooth transitions toward more sustainable and equitable futures ( Riedy & Waddock, 2022). However, such models frequently overlook the persistent conflicts, suffering and inequalities that shape the lived experiences of marginalized communities ( Joseph et al., 2021; Wittmer, 2020). Scholars argue that transformative social processes must directly engage with deeply ingrained inequalities and recognize that conflict and suffering are not merely obstacles but integral forces driving meaningful change ( August & Westphal, 2024; Boström et al., 2018; Bloomfield et al., 2006; Skrimizea et al., 2020; Wittmer, 2020).

Recent research highlights the transformative potential of conflict in addressing climate change and social inequalities. Ciplet and Harrison (2020) identify tensions between sustainability goals and justice concerns, emphasizing the need for inclusive governance processes. Nursey-Bray (2016) calls for a fundamental shift in understanding conflict, not as a cause-outcome relationship but as a dynamic force driving innovation and just adaptive governance. This perspective becomes particularly relevant in light of the COVID-19 pandemic, which has both exacerbated existing inequalities for Roma and Egyptian communities and exposed the tensions between dominant transitional justice narratives and the often-overlooked, ongoing struggles of these marginalized groups ( Dervishi et al., 2024). In Albania, recent historical developments and the broader political context further illuminate these hidden conflicts, revealing how systemic exclusion persists despite formal justice frameworks. Understanding these dynamics offers insight into how crises like COVID-19 amplify pre-existing vulnerabilities while simultaneously creating potential spaces for adaptive governance and systemic reform ( Council of Europe Office in Tirana, 2025).

Krause (2018) explores transformative approaches to climate change, emphasizing the importance of eco-social policies that prioritize sustainable and just outcomes. These approaches recognize that conflict, whether socio-political or environmental, can catalyze systemic change rather than merely being a challenge to overcome. Similarly, Valero et al. (2024) found that community-led initiatives and inclusive actions promote long-term resilience, illustrating how conflict-driven inequalities can inspire grassroots mobilization and institutional adaptation. In the context of Roma and Egyptian communities, these perspectives highlight how marginalized groups, despite facing structural injustices, actively engage in adaptive governance and resistance. By acknowledging the role of hidden conflicts—such as systemic discrimination, exclusion from public services, educational segregation, stigmatizing cultural norms, and the lack of genuine political representation—in shaping the experiences of Roma and Egyptian communities, justice mechanisms can shift from reactive measures to proactive strategies that foster meaningful, conflict-driven transformation. These conflicts often remain obscured within dominant narratives, bureaucratic procedures, and development rhetoric, yet they significantly influence the ongoing marginalization and exclusion of these groups.

Collectively, these studies underscore the urgency of conflict-driven transformation that directly addresses systemic inequalities and promotes climate justice, particularly for marginalized communities ( Ciplet & Harrison, 2020; Nursey-Bray, 2016; Valero et al., 2024; Krause, 2018). The relevance of conflict and suffering becomes particularly pronounced in contexts marked by systemic discrimination and social exclusion ( Stewart et al., 2006).

By examining these issues, this paper outlines the conceptual framework of conflict-driven transformation and its relevance to transitional justice. It then details the methodological rationale behind the case study selection, followed by an in-depth analysis of each case. The concluding section reflects on the broader implications for justice frameworks and policy interventions, emphasizing the need for adaptive governance that integrates conflict as a force for innovation and systemic change.

## 2. Theoretical Framework

This article develops a theoretical framework that brings conflict theory, agonistic pluralism, and the Capitalocene into dialogue to critically assess how processes of transformation can address deep-rooted inequalities and exclusions. While conflict theory and agonistic theory have individually contributed to understandings of societal change, their potential to inform just transition discourses, especially in contexts marked by persistent marginalization, remains largely unexplored. This article fills that gap by proposing a conflict-driven, agonistic interpretation of just transition, informed by a Capitalocene critique of capitalist environmental and social harm.

At the heart of critical theory lies the recognition that societal transformations emerge through confrontation with structural inequalities and hegemonic power. Conflict theory, rooted in Marxist thought, positions conflict as not only inevitable but necessary for challenging dominant institutions and ideologies. Rather than viewing conflict as a breakdown of order, it understands it as a revealing force—exposing systemic failures, making injustice visible, and enabling transformative possibility ( Fraser & Jaeggi, 2018; Avelino, 2017; Avelino et al., 2023).

This perspective also aligns with Gramsci’s theory of hegemony, where social order is maintained through the suppression of dissent and the construction of a shared narrative of consensus ( Gramsci, 1971; Lears, 1985). In this sense, transformation requires not only material redistribution but also the politicisation of dissent, conflict, and excluded voices.

Chantal Mouffe’s theory of agonistic pluralism is particularly useful for conceptualising how democratic politics can engage with rather than suppress conflict. Against deliberative models that seek rational consensus, Mouffe proposes that democracy thrives when it transforms antagonism into agonism—a space where adversaries acknowledge each other’s right to differ ( Mouffe, 2005). In contexts of exclusion, such as among Roma and Egyptian communities in Albania, this perspective offers an important entry point for theorising how silenced voices might enter and reshape political debate( Menga, 2017).

Critics of agonistic theory have noted its insufficient engagement with the economic and structural constraints shaping who gets to participate in democratic conflict ( August, 2022; Erman, 2009). Moreover, agonistic theory often under-theorises capitalism as a structuring force, treating political contestation somewhat in isolation from material conditions ( Mazur-Bubak, 2019). This article addresses this gap by bringing in the Capitalocene as a necessary supplement to agonistic theory.

The concept of the Capitalocene, as developed by Moore (2017), challenges dominant Anthropocene framings by arguing that environmental crises are not caused by “humanity” in general but by the historical and ongoing logic of capitalism. This critique is crucial for a deeper understanding of just transition because it situates ecological destruction and social exclusion as mutually reinforcing products of capitalist systems of accumulation, exploitation, and dispossession.

Framing transformation within the Capitalocene highlights how systemic inequalities, including racialised, economic, and political exclusions, are not aberrations but structural features of capitalist development. This is especially evident in the case of Roma and Egyptian communities, whose marginalisation cannot be separated from the historical political economy of urban planning, land dispossession, and exclusion from sustainability policies. The Capitalocene lens thus reinforces the idea that conflict and exclusion are not exceptional but expected outcomes of capitalist transitions, including those branded as “green” or “just” ( Fetting, 2020).

Moreover, the Capitalocene critique directly strengthens agonistic theory by exposing the limits of elite-driven deliberation in sustainability governance. While agonistic pluralism calls for open contestation, Capitalocene theory reminds us that not all conflicts are heard or legitimised within existing institutional frameworks, particularly when capitalist interests shape the boundaries of what counts as “reasonable” dissent.

Together, these three theoretical strands contribute to a more conflict-sensitive and justice-oriented framework for understanding transition. Conflict theory helps illuminate how power operates through exclusion and structural violence; agonistic theory offers a way to conceptualise conflict as a democratic force; and the concept of Capitalocene situates these dynamics within a broader critique of capitalist environmental governance.

This framework challenges dominant narratives of just transition that prioritise technocratic consensus or institutional reform without adequately addressing underlying systems of oppression. Instead, it argues for a re-politicisation of transformation—one that recognises the importance of agonistic conflict, engages with structural critique, and centres the voices and struggles of marginalised communities.

By applying this theoretical framework to the case of Roma and Egyptian communities in Albania, the article explores whether agonistic practices can meaningfully engage with systemic exclusion and whether the intersection of Capitalocene critique and agonistic pluralism offers a more robust basis for transformative justice. The analysis that follows highlights how conflict, far from being a disruption, is a vital mechanism through which excluded groups assert their rights, challenge dominant structures, and demand recognition in shaping their futures.

## 3. Socioeconomic challenges of Albania’s Roma and Egyptian communities

The marginalization of Roma and Egyptian communities in Albania is rooted in long-standing historical exclusions. Under the Ottoman Empire, both groups were restricted to stigmatized social and occupational roles ( Piasere et al., 2014). The communist regime introduced formal integration mechanisms, guaranteed housing, employment, and education, but these were largely superficial and failed to dismantle systemic prejudice ( De Soto et al., 2005). The 1990s transition to a market economy deepened these vulnerabilities: state industries collapsed, and many Roma and Egyptians were pushed into informal labor markets and impoverished urban peripheries ( UNDP, 2019).

Despite the adoption of multiple inclusion policies, such as the *National Action Plan for Integration of Roma and Egyptians (2015–2020)* and the *Social Inclusion Policy Document (2021–2025)*, persistent disparities in education, employment, and housing reveal the limitations of consensus-driven governance ( Government of Albania, 2021). These frameworks tend to treat inclusion as a technocratic issue solvable through policy design, rather than as a deeply political struggle rooted in power asymmetries ( Ciplet & Harrison, 2020; Miconi et al., 2023). Inclusion efforts often prioritize institutional stability and national image (especially in the context of EU accession) over confronting ( Council of Europe Office in Tirana, 2025) entrenched social hierarchies. As such, they reflect a consensus model that avoids political conflict, treats marginalization as a management problem, and sidelines the agency of the affected communities.

Education remains a primary site of exclusion. Policies like the *National Action Plan for Integration* have not prevented disproportionately high dropout rates among Roma and Egyptian children ( European Commission, 2018; Miconi et al., 2023). Factors such as poverty, bullying, and the absence of culturally responsive pedagogy contribute to their disengagement ( Miconi et al., 2021; Dervishi et al., 2022a; UNDP, 2019, 2025a, 2025b). These students often experience school segregation and discrimination practices that persist despite formal inclusion mandates. This outcome reveals that consensus-based reforms, although well-intentioned, fail to address institutional racism and structural barriers ( Dervishi et al., 2022b). They often rely on top-down administrative strategies without engaging with the lived realities and demands of Roma and Egyptian families.

Employment prospects for these communities are similarly constrained. Roma and Egyptians are disproportionately concentrated in the informal economy, performing unstable and low-paid work without social protections ( UNDP, 2019). This is not merely a consequence of market forces, but of policy frameworks that have failed to dismantle systemic discrimination in access to formal employment and vocational training ( De Soto et al., 2005). Albania’s post-socialist economic transition embraced neoliberal growth without redistributive safeguards, exacerbating inequality along racialised and classed lines.

Housing conditions further reflect structural neglect. Many Roma and Egyptian families live in informal settlements lacking secure tenure, sanitation, or legal protection against eviction ( Open Society Foundation for Albania, 2014; UNDP, 2019). Housing strategies have largely focused on regularization or relocation, but implementation has been inconsistent, underfunded, and disconnected from community participation. The result is a continued pattern of displacement without redress, often under the guise of urban development ( United Nations Development Programme, 2019). Again, the consensus-oriented nature of these policies, designed to demonstrate compliance with international standards, tends to paper over deeper power inequalities and fails to involve affected communities in shaping outcomes.

While these inclusion strategies may appear progressive, they reflect a technocratic consensus model that seeks to avoid political confrontation, thereby reinforcing rather than resolving exclusion. The theoretical framework proposed in this article, drawing on agonistic pluralism and critical theories of the Capitalocene, argues that meaningful transformation requires engaging with conflict, not avoiding it. The failure of consensus-based reforms suggests that structural change emerges through struggle, contestation, and the politicization of inequality.

In contrast to the failure of consensus-oriented approaches, recent examples of agonistic interventions demonstrate the political agency of marginalized communities and the potential of conflict to disrupt exclusionary systems.

A landmark instance is the European Court of Human Rights (ECHR) ruling on June 1, 2022, in favor of five Roma and Egyptian families who challenged the ethnic segregation of their children at the ‘Naim Frashëri’ elementary school in Korçë. With support from the European Roma Rights Centre, the families bypassed domestic legal systems and filed the case internationally, claiming a violation of Article 1, Protocol 12 of the European Convention on Human Rights. The ECHR concluded that Albania had failed to take effective desegregation measures and upheld systemic racial discrimination in education ( Gazeta Shqiptare, 2022).

This case exemplifies agonistic politics in Chantal Mouffe’s sense: political confrontation with state structures becomes a vehicle for democratic renewal. The success of the case lay not only in the ruling itself, whose long-term effects remain to be seen, but in the act of contestation, which disrupted Albania’s self-narrative of inclusive governance. It forced the state, and by extension, EU observers, to confront the gap between policy rhetoric and lived experience. In this sense, the case pushed for social change by shifting public discourse and legitimizing Roma and Egyptian claims in international law.

A second example of agonistic mobilisation occurred during the COVID-19 pandemic, when Roma and Egyptian communities organized protests against their exclusion from emergency food aid. While state narratives emphasized universal assistance, protestors revealed that many Roma and Egyptians were systematically left out due to a lack of documentation, discriminatory distribution practices, and bureaucratic inaccessibility ( Dervishi et al., 2022b). These actions, although small in scale, succeeded in drawing media attention and pressuring local governments to acknowledge their failure.

These examples, legal action and public protest, show that conflict is not a breakdown of democratic order but a sign of its vitality. Agonistic interventions challenge the myth of neutrality underpinning technocratic inclusion policies and expose the structural violence that such policies obscure. As Albania aspires to join the EU, these conflicts highlight the importance of institutionalizing dissent and recognizing that justice requires more than policy—it requires political confrontation and community-led resistance.

## 4. The role of conflict and suffering in transformation

Social transformation is often catalyzed by conflict, particularly when marginalized communities confront entrenched inequalities and systemic exclusions. Rather than a breakdown of social order, conflict is increasingly understood as an engine of change. Critical theorists like Fraser (2018) argue that agonistic contestation plays a crucial role in exposing injustices and initiating structural redress. In this framework, suffering becomes politically productive when it translates into visible contestation, making inequality not only legible but also challengeable.

In Albania, Roma and Egyptian communities have long experienced exclusion from housing, employment, and education. Their resistance has manifested through both legal mechanisms and street-level protest, exemplifying the productive dimensions of conflict. For instance, in 2018, Roma families in Shkodra faced forced eviction from long-standing settlements. Activists, supported by NGOs, responded with protests and legal actions, resulting in temporary injunctions that suspended the evictions. Though only a partial victory, this case illustrates how conflict can force institutional recognition of marginalized voices and prompt re-engagement with neglected issues like housing security ( Open Society Foundation for Albania, 2014).

The COVID-19 pandemic deepened these existing inequalities. Roma and Egyptian communities were among the hardest hit, facing loss of income, access to food, and health services. A report by Kryeziu (2020) vividly captured the extent of their suffering:

*“There is not a single crumb of bread in their homes, their children are crying for food… These families will be seriously threatened by hunger.”*

Crucially, this suffering was not met with silent endurance. In the cities of Fier and Elbasan, members of these communities protested in the streets, demanding food aid and government support. These protests exemplify *agonistic conflict*: expressions of suffering that assert political agency and demand recognition. They also reflect how suffering, when made visible through public demonstration, can disrupt the dominant narratives of crisis management and force attention to structural neglect. These food aid protests thus serve as clear examples of agonism at work, underscoring the transformative potential of suffering-driven mobilization.

A recurring pattern emerges from these episodes: marginalized communities often rely on *non-governmental institutions*, local NGOs, activist networks, and community leaders to amplify their struggles and provide basic support. In the absence of responsive state intervention, these institutional “crutches” ( Annist, 2015) play a crucial intermediary role in transforming suffering into recognized claims.

This dynamic was evident after the 2018 flooding. While the Albanian government provided only temporary relief, failing to address the root causes, such as poor drainage and discriminatory urban planning, NGOs partnered with Roma leaders to mobilize aid and push for infrastructure reform ( European Commission, 2018). These initiatives, though constrained, represent grassroots-led responses that navigate and contest systemic exclusion. Such patterns raise critical questions: Does meaningful transformation for suffering communities require the mediation of institutional allies? Suffering alone does not drive transformation unless scaffolded by institutions capable of amplifying, legitimizing, or channeling it. This echoes broader debates in democratic theory and ethics of care, where the “institutionalization of suffering” ( Stowell & Warren, 2018) is necessary to move from private pain to public redress.

The food protests and post-flood responses also highlight a key distinction: the primary actors enabling transformation are often non-state institutions. The Albanian state, in both crises, remained reactive, managerial, and focused on short-term relief. In contrast, NGOs and grassroots activists responded more proactively, pushing for deeper structural solutions. For example, after the 2018 floods, local NGOs collaborated with Roma activists to provide continued educational support to displaced children. This intervention helped maintain access to education while emphasizing the need for long-term investment as a pathway out of poverty ( World Bank, 2019). Such efforts illustrate how community-based responses can create *incremental but durable* improvements, even when state engagement is minimal or absent.

Yet, as Fraser ( McManus, 2020) notes, resilience and self-help, while admirable, are not substitutes for structural transformation. Without systemic change, addressing land inequality, urban exclusion and political underrepresentation, grassroots efforts remain palliative rather than transformative.

These events also intersect with broader questions of environmental justice. The Capitalocene framework ( Moore, 2017) shifts the focus from generic human responsibility to the systemic exploitation characteristic of capitalist development. In this light, Albania’s marginalized communities face *compound injustices*: they are both economically exploited and ecologically exposed. Environmental degradation, such as flooding and inadequate sanitation, impacts Roma communities more severely due to their housing precarity. Yet their suffering remains largely invisible in mainstream environmental discourse.

As Ciplet and Harrison (2020) argue, the fight for environmental justice must center those most impacted. When local actors mobilize, cleaning flood-damaged areas, petitioning municipalities, or demanding infrastructure, they enact an agonistic environmentalism that contests both state neglect and global environmental injustice.

In Albania, that means recognizing Roma and Egyptian communities not just as victims but as agents of transformation—actors whose suffering, when amplified by conflict and institutional support, can disrupt dominant paradigms and call for systemic change.

## 5. Critical analysis of just transition policies

Just Transition concerns are often highlighted in political discussions, but tend to overlook the realities of oppressed groups and communities ( Bueno Patin & Stapper, 2025). Rhetoric centered on fairness suggests that with a Just Transition no one will be left behind; however, there is an enormous chasm between the outcomes experienced and justice. This gap is particularly striking in economically transitioning regions such as Albania, which incorporate Just Transition policies in their frameworks.

Disregarded social issues arise from focusing on Just Transitions infrastructures as their primary framework ( Butler et al., 2016). This gap remains at the core of Why Toils refuse to acknowledge alongside Schlosberg underlines this matter ( 2007): “the gap between idealized policy rhetoric and the lived realities of disadvantaged communities.” In nearly all cases, women, ethnic minorities or low-income earners advertising adequate social policies face multi-dimensional discrimination due to an insufficient green discourse providing merely timid digits. “Mainstream” does not approach these problems in any reliable manner.

Equally suppressive is trying to implement greener goals-planned obsolescence or change economic structure capitalism parallel within transition focused on socio-environmental growth ( Piggot *et al., *2019). For example, retraining programs for ex-coal miners often overlook the informal sectors where Roma, Egyptians, and other marginalized groups work ( Qejvanaj, 2021). Such policies are designed without considering the socio-economic and cultural realities of marginalized communities. These policies do prominent harm by deepening inequalities instead of addressing them.

A prime example is Albania’s National Action Plan for Integration of Roma and Egyptians which epitomizes how Just Transition frameworks can either be poorly enacted or completely bypass marginalized populations. While the plan does contain strategic social inclusion goals related to employment, education, and health, its actual reach and outcomes for the Roma and Egyptian communities have been scant ( European Commission, 2018). The Council of Europe (2025) illustrates this by stating “the persistent gap between Roma and non-Roma populations in terms of living standards, employment rates, and education remains a significant challenge.”

The absence flexible funding streams coupled with entrenched systemic barriers means that integrated approaches to national transitions—such as green jobs or skill development training—tend to exclude Roma and Egyptians.

Additionally, the lack of Roma and Egyptian cultural awareness and intersectionality considerations results in sidelining their concerns. Without marginalized groups actively participating in policymaking processes, meaningful change becomes impossible.

The attempts to integrate Roma and Egyptian communities through national policies seem to reflect a consensus-oriented policy framework. Governments, international bodies, or non-government organizations advocate for inclusion with hopes of effortless transitions accompanied by economic prosperity and social equality ( Tørres, 2021). But often these attempts are rejected by the very marginalized communities at which they are aimed. The issues that these communities face go beyond just structural; they stem from enduring cultural and social backlash against imposed solutions — this is where conflict surfaces ( Ullman & Kittner, 2024). It is precisely the people who are intended to benefit from these policies who resist the seamless integration suggested by policymakers because such approaches fail to consider their real challenges.

Fostering diverging opinions while accepting genuine conflicts that emerge when policies disregard the realities of life for marginalized groups engage is what defines agonistic approaches to Just Transition.

This perspective highlights the pivotal role of conflict during political activity and that society change happens as a result of struggle ( Truth and Reconciliation Commission of Canada, 2015). Instead of focusing on consensus only, this approach appreciates grappling with conflicts that emerge when policies diverge from the needs of marginalized peoples. One clear illustration is some lawsuits brought to ECHR for women’s rights. Those are what we call agonistic responses. These legal battles are not solely aimed at attaining justice for some wrongs; they aim to contest systemic socially entrenched capitals inequities.

By dragging these issues to court, the affected populations initiate a process in which their ignored policies have to be reconsidered at least on the surface level due to claimsmaking politics. While doing so, such groups challenge unworthy institutional frameworks without paying attention or subscribing to whatever decision is made in return as an act of sovereignty. Such forms of dissent cannot be characterized predominantly as oppositional, because they also enrich important social discussions.

Active policies cause intervener government and institutions rejection or modification. In other words, these shifts break ritualistic republicanism where there is false acceptance masking unwillingness to deal with sore absentees from public life and obliterate silence inflicted by active denial against excluded subjects.

Fostering an inclusive political structure that helps achieve a Just Transition requires amplification of vulnerable groups’ dissenting voices to be heard. Marginalized communities not only seek change through protests and legal actions, but they also create opportunities for meaningful discussions regarding social justice. In the struggle for representation and inclusion, these communities transform unjust systems by fighting to reshape policies toward greater societal needs.

Efforts toward climate justice can benefit from a balance between consensus approaches like government policies, NGO initiatives, and agonistic approaches like protests and community resistance. Addressing conflictual processes makes Just Transitions responsive to the most vulnerable populations.

## 6. Pathways for justice-oriented transitions

Over time, justice has become a critical concern in the context of sustainability transitions, assessed both as an objective and as a framework within which to consider their governance and legitimacy. In the context of “Capitalocene,” which shifts attention from Anthropocene blame to capitalism’s violent systemic-ecological world regime, the demand for justice goes beyond distributional equity. It fundamentally requires transformation and reparative action along with thoroughly confronting inequality, exploitation, and environmental harm’s sociohistorical roots. For these reasons, justice-focused transitions must shift from mere technical fixes to comprehensive changes that dismantle the dominant systems of accumulation, extraction, and dispossession.

As such, the notion of Just Transition serves as an essential starting framework. Developed within labor activism to mitigate economic restructuring impacts on workers, Just Transition now also includes environmental ( Goodman, 2018) and climate justice ( Harper et al., 2009) along with intersectional race, gender, ( Keddie, 2012) and coloniality-based concerns ( Martínez-Alier, 2020). But more recently it has been highlighted (e.g., McCauley & Heffron, 2018) that Just Transition needs to be placed under other more encompassing theoretical frames that integrate restorative, procedural logics alongside recognition-based and epistemic forms of justice.

Instead of considering restorative justice as an addition or enhancement of Just Transition, we suggest viewing it as a profound complement that integrates both agonistic and deliberative approaches to democratic participation.

An effective transition grounded on justice principles requires simultaneously reallocating resources, recognizing past injustices, and restructuring engagement. The inclusion and empowerment of historically marginalized and disenfranchised groups as active agents of change is vital for this process. Policy-making informed by participatory recognition-based justice grants agency to those most affected by the climate and ecological crises dominantly shapes the transition.

Agyeman et al. (2012) stress that environmental justice must go beyond distribution to include participatory and procedural dimensions. Citizens’ assemblies are often celebrated for their deliberative inclusivity; however, these formats may inadvertently reproduce some inequalities they set out to dismantle. In contexts characterized by stark power disparities, the drive for consensus can become a means to exclude, silence dissenting voices or stall transformative efforts. A justice-oriented pathway should focus on balancing deliberation with more agonistic forms of politics that embrace contestation, resistance and conflict as essential elements of democracy.

Addressing such fundamentally entrenched injustice is crucial because no amount of dialogue can negotiate them away.

Marginalized groups have historically relied on protest, lawsuits, and other forms of direct action to confront systemic injustice. These actions should be recognized as part the transition architecture framework, rather than being written off as fringe antagonistic interruptions. Such agonistic methods challenge the structural reasoning driving the Capitalocene and force transitions beyond pure technocratic concession.

In this regard, we advocate for a triadic framework of justice that consists of three interconnected aspects: first, inclusion-based policymaking that allows access and participation in all groups around the given structures to achieve distributive and procedural reforms; second, conflictual systems that allow constructional dispute disagreement space. These mechanisms recognize conflict and dissent as vital elements for democratic change; third, practices which strive to correct injury by accepting, repairing, and reconciling ‘doing wrong’ through intentional acts of undoing harm or injustice counteraction describes restorative injustices.

Zehr (2002) defines restorative justice as enabling a collective reckoning which permits communities to articulate harm, determine accountability, and collaboratively establish means of reparation. This is crucial in the context of climate injustice for Indigenous peoples and other ethnically marginalized groups who are descendants from long colonial resource-raping systems alongside epistemic erasure. Restorative frameworks can also fuel broader systemic change; Canada’s Truth and Reconciliation Commission is an example of this.

Crucially not engaging in conflict does not mean accepting defeat when practicing restorative justice.

Still, both can work together: While some practices disrupt dominant power configurations, other restorative approaches reweave fractured social relations and cultivate conditions for enduring reconciliation. Collectively, they expand the democratic horizons of transition processes and allow conflict as well as compassion, dissent alongside repair.

Thus, justice transitions need participation modes like activism, protest, deliberation and storytelling tooperate within a single system. According to Escobar (2016) and Mansbridge et al. (2012), democracy in complex systems requires polycentric formal and informal civic structures with systemic functions.

The situated nature of justice claims is illustrated further in case studies. In South Africa, Just Transition efforts within the coal sector have emphasized direct compensation along with oversight mechanisms which has stemmed from historically low trust in the state ( Wilgosh et al., 2022). On the other hand, African-led initiatives elsewhere on the continent are focused more on community-led deliberation alongside local knowledge ( Otlhogile & Shirley, 2023). These examples emphasize plural contextual frameworks that are responsive to culture history institutional specificity.

In the end, a just transition cannot be framed only from one angle; it involves multiple perspectives reflecting a collective re-envisioning of managing change while fundamentally redefining who determines its terms autodetermination.

Examining the ideas of Capitalocene, agonistic democracy, and restorative action, we can place justice more at the center of our theory and practice, enabling us to work toward transitions that are sustainable and emancipatory.

## 7. Conclusion

This article contributes to the growing body of scholarship on justice-oriented transitions by emphasizing the transformative potential of conflict, particularly for marginalized communities. By focusing on the experiences of Albania’s Roma and Egyptian populations, it illustrates how conflict-driven change can illuminate the deep inequalities embedded in climate justice discourses and practices.

The findings underscore that many current Just Transition policies, despite their rhetorical commitment to equity, often fail to meaningfully engage marginalized groups. This is especially evident in cases such as Albania’s National Action Plan for Roma and Egyptians, where well-intentioned policy frameworks have not translated into inclusive outcomes ( European Commission, 2018). The disconnect between policy ambition and lived reality highlights the insufficiency of transition processes that prioritize technocratic consensus over political contestation.

A key insight of this analysis is the inadequacy of consensus-based approaches that dominate mainstream Just Transition discourse. Such approaches often depoliticize transition by seeking to neutralize dissent and obscure structural inequalities. Drawing on Mouffe’s theory of agonistic pluralism, this article argues for the necessity of institutionalizing dissent rather than suppressing it. Agonistic approaches recognize conflict as a generative force for democratic renewal and justice. In this context, dissent becomes a mechanism for marginalized communities to assert political agency and demand structural change.

This perspective aligns with Swyngedouw’s (2011) critique of post-democratic governance, which cautions against the foreclosure of egalitarian political spaces under the guise of technocratic management. Transition governance that sidelines dissent reproduces the same capitalist and colonial logics that underpin the Capitalocene. Within this frame, the experiences of Roma and Egyptian communities in Albania reflect broader dynamics in which climate and sustainability transitions risk entrenching the hegemonic systems of racialized and class-based exclusion.

From a conflict theory standpoint, the struggles of these communities highlight the need to politicize Just Transition processes by foregrounding issues of power, historical harm and redistribution. Pellow (2023) argues that conflict recognition is essential to address past injustices and for preventing their reproduction. This means acknowledging the antagonisms inherent in environmental politics and resisting the urge to resolve them through depoliticized, top-down solutions.

Achieving a truly just transition requires transformative governance approaches that recognize conflict as an opportunity for systemic change. Practices such as participatory planning, community-based climate action, and participatory budgeting can institutionalize dissent by creating spaces where marginalized voices are central to decision-making ( Agyeman et al., 2012). These mechanisms, however, must be coupled with material investments in disadvantaged communities and the dismantling of institutionalized discrimination in labor markets, housing, and environmental regulation.

Restorative justice and healing practices also offer pathways for redress. Recognizing the socio-emotional injuries of systemic exclusion through reconciliation processes, cultural revitalization, and recognition of community knowledge can empower historically marginalized groups and foster solidarity across difference ( Zehr, 2002; Plumwood, 2012).

In conclusion, the path toward a Just Transition must be conflict-aware, historically grounded, and structurally transformative. By drawing on conflict theory, the critique of the Capitalocene, and agonistic political theory, researchers argue for a model of transition that centers dissent as a democratic force. Such a model does not fear conflict but embraces it as a vital driver of social and ecological transformation. Institutionally supporting rather than neutralizing such dissent can create transition processes that dismantle entrenched power relations and build genuinely inclusive and sustainable futures.

## Ethics and consent

Ethical approval and consent were not required.

The research is based exclusively on qualitative policy analysis and the review of publicly available documents and academic literature. It did not involve human participants, personal data, or primary data collection.

## Data Availability

This study is based on qualitative policy analysis and a critical review of publicly available policy documents and academic literature. No primary empirical data were generated or analysed during this study. All documents cited are publicly accessible through official institutional websites and the sources listed in the reference section. As the article does not involve original quantitative or qualitative datasets, there are no underlying data (e.g., raw datasets, numerical values, or image data) to deposit in a public repository.
