# From marginalization to transformation: Examining structural injustices and policy impacts on Albania’s Roma and Egyptian communities

**DOI:** 10.12688/openreseurope.23119.2

**Published:** 2026-07-11

**Authors:** Eglantina Dervishi

**Affiliations:** 1 Universiteti i Tiranes Fakulteti i Shkencave Sociale, Tirana, Tirana County, Albania

**Keywords:** conflict-driven transformation, environmental justice, marginalized communities, power dynamics, socioeconomic exclusion, sustainable transitions

## Abstract

This study explores the role of conflict, suffering, and structural injustices in driving transformative processes within Albania’s Roma and Egyptian communities, emphasizing how these experiences challenge consensus-driven approaches to social change. By analyzing systemic inequalities and power struggles through critical theory frameworks, the study highlights the importance of addressing conflict as a catalyst for transformation in the face of global challenges like climate change and the Capitalocene. The research draws on policy reviews and academic literature to examine the intersection of social exclusion, poverty, and environmental justice, revealing the limitations of current ‘just transition’ policies. The findings underscore that conflict and inequality must be acknowledged and addressed to achieve meaningful, justice-oriented transitions, particularly in marginalized communities.

## 1. Introduction

This article aims to contribute to the growing body of scholarship that explores conflict as a key driver of social and environmental transformation. By centering on the experiences of Albania’s Roma and Egyptian communities, it reveals the broader implications of conflict-driven change within the context of climate justice and systemic inequality. Through a critical review of academic literature and policy documents, and by linking local struggles to global challenges, the article underscores the necessity of embedding conflict into governance and policy frameworks as a catalyst for meaningful and lasting transformation.

Transformation processes are often framed within dominant narratives of consensus, harmony and prosperity, emphasizing smooth transitions toward more sustainable and equitable futures ( Riedy & Waddock, 2022). However, such models frequently overlook the persistent conflicts, suffering and inequalities that shape the lived experiences of marginalized communities ( Joseph et al., 2021; Wittmer, 2020). Scholars argue that transformative social processes must directly engage with deeply ingrained inequalities and recognize that conflict and suffering are not merely obstacles but integral forces driving meaningful change ( August & Westphal, 2024; Boström et al., 2018; Bloomfield et al., 2006; Skrimizea et al., 2020; Wittmer, 2020).

Recent research highlights the transformative potential of conflict in addressing climate change and social inequalities. Ciplet and Harrison (2020) identify tensions between sustainability goals and justice concerns, emphasizing the need for inclusive governance processes. Nursey-Bray (2016) calls for a fundamental shift in understanding conflict, not as a cause-outcome relationship but as a dynamic force driving innovation and just adaptive governance. This perspective becomes particularly relevant in light of the COVID-19 pandemic ( Dervishi et al., 2021), which has both exacerbated existing inequalities for Roma and Egyptian communities and exposed the tensions between dominant transitional justice narratives and the often-overlooked, ongoing struggles of these marginalized groups ( Dervishi et al., 2024). In Albania, recent historical developments and the broader political context further illuminate these hidden conflicts, revealing how systemic exclusion persists despite formal justice frameworks. Understanding these dynamics offers insight into how crises like COVID-19 amplify pre-existing vulnerabilities while simultaneously creating potential spaces for adaptive governance and systemic reform ( Council of Europe Office in Tirana, 2025).

Krause (2018) explores transformative approaches to climate change, emphasizing the importance of eco-social policies that prioritize sustainable and just outcomes. These approaches recognize that conflict, whether socio-political or environmental, can catalyze systemic change rather than merely being a challenge to overcome. Similarly, Valero et al. (2024) found that community-led initiatives and inclusive actions promote long-term resilience, illustrating how conflict-driven inequalities can inspire grassroots mobilization and institutional adaptation. In the context of Roma and Egyptian communities, these perspectives highlight how marginalized groups, despite facing structural injustices, actively engage in adaptive governance and resistance. By acknowledging the role of hidden conflicts—such as systemic discrimination, exclusion from public services, educational segregation, stigmatizing cultural norms, and the lack of genuine political representation—in shaping the experiences of Roma and Egyptian communities, justice mechanisms can shift from reactive measures to proactive strategies that foster meaningful, conflict-driven transformation. These conflicts often remain obscured within dominant narratives, bureaucratic procedures, and development rhetoric, yet they significantly influence the ongoing marginalization and exclusion of these groups.

Collectively, these studies underscore the urgency of conflict-driven transformation that directly addresses systemic inequalities and promotes climate justice, particularly for marginalized communities ( Ciplet & Harrison, 2020; Nursey-Bray, 2016; Valero et al., 2024; Krause, 2018). The relevance of conflict and suffering becomes particularly pronounced in contexts marked by systemic discrimination and social exclusion ( Stewart et al., 2006).

By examining these issues, this paper outlines the conceptual framework of conflict-driven transformation and its relevance to transitional justice. It then details the methodological rationale behind the case study selection, followed by an in-depth analysis of each case. The concluding section reflects on the broader implications for justice frameworks and policy interventions, emphasizing the need for adaptive governance that integrates conflict as a force for innovation and systemic change.

## 2. Methodology

### 2.1 Study design

This study adopts a qualitative, interpretive approach based on the analysis of documents. Rather than collecting primary data, it draws on existing sources to explore how conflict, structural inequality, and processes of transformation are understood in relation to Roma and Egyptian communities in Albania. The analysis is informed by the theoretical framework outlined in the paper, which guides both the selection of materials and their interpretation.

### 2.2 Selection of sources

The study focuses on sources that are directly relevant to the Albanian context or to similar regional dynamics. These include academic publications, policy documents, and reports produced by national and international institutions. Priority was given to materials that address issues such as social exclusion, inequality, governance, and Just Transition policies.

Sources that were purely descriptive, lacked analytical depth, or were not clearly connected to the research focus were not included. This helped ensure that the analysis is grounded in material that is both relevant and informative.

### 2.3 Data collection strategy

A purposive approach was used to identify key sources. Rather than aiming for exhaustive coverage, the goal was to select materials that are particularly relevant for understanding the structural dynamics under study.

Sources were identified through searches in academic databases such as Scopus and Google Scholar, as well as through the review of institutional and policy documents from organizations such as the European Commission and UNDP. In addition, reference lists of key publications were used to locate further relevant studies.

Most of the sources consulted were published between 2010 and 2024, in order to reflect current developments, while earlier works were included where necessary to provide historical context.

### 2.4 Analytical approach

The analysis combines thematic reading with elements of critical discourse analysis. First, the material was reviewed to identify recurring themes, particularly in relation to patterns of inequality, policy approaches, and forms of conflict and resistance.

At the same time, attention was paid to how key issues—such as inclusion, marginalization, and “Just Transition”—are framed in policy and institutional narratives. This made it possible to examine not only what is said, but also how certain perspectives are emphasized or overlooked.

The process was iterative, involving repeated reading and refinement of themes. Throughout, the theoretical framework served as a guide, ensuring that the analysis moves beyond description and engages with broader questions of power, conflict, and structural inequality.

### 2.5 Use of case examples

To strengthen the connection between theory and empirical reality, the study draws on selected examples, including the European Court of Human Rights ruling on school segregation and the protests that took place during the COVID-19 pandemic.

These cases were not chosen to provide a comprehensive account, but rather to illustrate how structural inequalities become visible and contested in practice. They serve as concrete entry points for understanding how conflict operates within the broader processes discussed in the paper.

### 2.6 Rigor and limitations

Several steps were taken to ensure the credibility of the analysis, including the use of clearly defined selection criteria and the combination of different types of sources (academic, policy, and institutional). The consistent use of the theoretical framework also helps maintain coherence across the analysis.

At the same time, the study has limitations. It relies entirely on secondary data and does not include primary research or direct accounts from community members. In addition, the purposive selection of sources means that the analysis is not exhaustive. However, these choices are consistent with the aim of the paper, which is to provide a theory-informed interpretation rather than a comprehensive empirical study.

## 3. Theoretical framework

This article develops a theoretical framework that brings conflict theory, agonistic pluralism, and the Capitalocene into a complementary and analytically distinct relationship, in order to assess how transformation processes can address deep-rooted inequalities and exclusions. Rather than treating these approaches as parallel discussions, the framework assigns each a specific role: conflict theory explains the structural production of inequality, agonistic pluralism conceptualizes the democratic expression of conflict, and the Capitalocene situates both within a broader critique of capitalist development.

While these perspectives have individually contributed to understandings of societal change, their combined application to just transition debates in contexts of persistent marginalization remains limited. This article addresses this gap by proposing a conflict-driven and empirically grounded interpretation of just transition.

At its core, conflict theory emphasizes that societal transformation emerges through confrontation with structural inequalities and power asymmetries. Rooted in Marxist thought, it positions conflict as not only inevitable but necessary for challenging dominant institutions and ideologies. Rather than viewing conflict as a breakdown of order, it understands it as a revealing force—exposing systemic failures, making injustice visible, and enabling transformative possibility ( Fraser & Jaeggi, 2018; Avelino, 2017; Avelino et al., 2023). In the Albanian context, this perspective helps explain how persistent disparities in education, employment, and housing among Roma and Egyptian communities are not accidental, but structurally produced and maintained over time.

This perspective also aligns with Gramsci’s theory of hegemony, where social order is maintained through the suppression of dissent and the construction of a shared narrative of consensus ( Gramsci, 1971; Lears, 1985). From this standpoint, consensus-driven inclusion policies can be interpreted not as neutral solutions, but as mechanisms that stabilize existing power relations by depoliticizing inequality. Transformation therefore requires not only redistribution, but also the politicisation of dissent and the visibility of marginalized claims.

Building on this, Chantal Mouffe’s theory of agonistic pluralism provides a framework for understanding how conflict can be productively incorporated into democratic processes. Against deliberative models that prioritize rational consensus.

Mouffe argues that democracy thrives when antagonism is transformed into agonism—a condition in which conflicting groups engage as legitimate adversaries ( Mouffe, 2005).

This perspective is particularly relevant in the Albanian case, where instances such as the European Court of Human Rights ruling on school segregation and COVID-19-related protests illustrate how marginalized communities actively contest exclusion through legal and political means ( Menga, 2017). These cases demonstrate that conflict is not external to democratic systems, but a central mechanism through which excluded groups gain visibility and influence. However, agonistic theory has been criticized for insufficiently addressing the structural and economic conditions that shape participation ( August, 2022; Erman, 2009).

It often treats political contestation in relative isolation from material inequalities ( Mazur-Bubak, 2019). To address this limitation, the present framework incorporates the concept of the Capitalocene, which reintroduces structural political economy into the analysis of conflict and transformation.

The Capitalocene, as developed by Moore (2017), challenges Anthropocene narratives by locating environmental and social crises within the historical logic of capitalism.

This perspective emphasizes that ecological degradation and social exclusion are co-produced through systems of accumulation, exploitation, and dispossession. In the Albanian context, this lens helps explain why Roma and Egyptian communities are disproportionately exposed to environmental risks (e.g., inadequate housing, flooding) while simultaneously excluded from the benefits of sustainability policies. By integrating the Capitalocene, the framework highlights that inequalities are not peripheral but structural outcomes of capitalist development, including those embedded within “green” or “just” transition policies ( Fetting, 2020).

It also strengthens agonistic theory by demonstrating that not all conflicts are equally recognized: institutional frameworks often privilege certain forms of participation while marginalizing others, particularly those that challenge dominant economic interests.

Taken together, these three theoretical strands form a coherent and non-redundant analytical framework. Conflict theory identifies how inequalities are produced and reproduced; agonistic pluralism explains how these inequalities become politically contested; and the Capitalocene situates both processes within a broader system of capitalist governance. This integration allows the framework to move beyond abstract theorization and directly engage with empirical realities. Importantly, the framework is not presented as a purely conceptual exercise but as a tool for interpreting empirical dynamics. For example, the ECHR desegregation case can be understood as an agonistic intervention that exposes structural inequalities identified by conflict theory, while also revealing the institutional limits emphasized by the Capitalocene critique. Similarly, COVID-19 protests by Roma and Egyptian communities illustrate how material deprivation (conflict theory) becomes politicized through public contestation (agonistic pluralism), within a context shaped by systemic economic exclusion (Capitalocene).

This framework challenges dominant narratives of just transition that prioritise technocratic consensus or institutional reform without adequately addressing underlying systems of oppression. Instead, it argues for a re-politicisation of transformation—one that recognises the importance of agonistic conflict, engages with structural critique, and centres the voices and struggles of marginalised communities.

By applying this framework to the case of Roma and Egyptian communities in Albania, the article demonstrates that conflict is not a disruption of transition processes, but a central mechanism through which marginalized groups articulate claims, challenge exclusion, and reshape governance structures.

## 4. Socioeconomic challenges of Albania’s Roma and Egyptian communities

The marginalization of Roma and Egyptian communities in Albania is rooted in long-standing historical exclusions.

To better capture the structural continuity of these processes, this section adopts a chronological and path-dependent perspective linking historical conditions to present inequalities. Under the Ottoman Empire, both groups were restricted to stigmatized social and occupational roles ( Piasere, 2014), which institutionalized forms of socio-economic exclusion that continue to shape patterns of labor market marginalization today. The communist regime introduced formal integration mechanisms, guaranteeing housing, employment, and education, but these were largely superficial and failed to dismantle systemic prejudice ( De Soto et al., 2005). While this period reduced extreme poverty through state provision, it did not eliminate underlying social hierarchies, resulting in fragile inclusion dependent on state structures. The 1990s transition to a market economy deepened these vulnerabilities: state industries collapsed, and many Roma and Egyptians were pushed into informal labor markets and impoverished urban peripheries ( UNDP, 2019). This transition represents a critical rupture, where the removal of state guarantees disproportionately affected already marginalized groups, reinforcing cumulative disadvantage across generations.

Despite the adoption of multiple inclusion policies, such as the *National Action Plan for Integration of Roma and Egyptians (2015–2020)* and the *Social Inclusion Policy Document (2021–2025)*, persistent disparities in education, employment, and housing reveal the limitations of consensus-driven governance ( Government of Albania, 2021). These frameworks tend to treat inclusion as a technocratic issue solvable through policy design, rather than as a deeply political struggle rooted in power asymmetries ( Ciplet & Harrison, 2020; Miconi et al., 2023). Inclusion efforts often prioritize institutional stability and national image (especially in the context of EU accession) over confronting ( Council of Europe Office in Tirana, 2025) entrenched social hierarchies. As such, they reflect a consensus model that avoids political conflict, treats marginalization as a management problem, and sidelines the agency of the affected communities.

Education remains a primary site of exclusion. Policies like the *National Action Plan for Integration* have not prevented disproportionately high dropout rates among Roma and Egyptian children ( European Commission, 2018; Miconi et al., 2023). Factors such as poverty, bullying, and the absence of culturally responsive pedagogy contribute to their disengagement ( Miconi et al., 2021; Dervishi et al., 2022a; UNDP, 2019, 2025a, 2025b). These students often experience school segregation and discrimination practices that persist despite formal inclusion mandates. This outcome reveals that consensus-based reforms, although well-intentioned, fail to address institutional racism and structural barriers ( Dervishi et al., 2022b). They often rely on top-down administrative strategies without engaging with the lived realities and demands of Roma and Egyptian families.

Employment prospects for these communities are similarly constrained. Roma and Egyptians are disproportionately concentrated in the informal economy, performing unstable and low-paid work without social protections ( UNDP, 2019). This is not merely a consequence of market forces, but of policy frameworks that have failed to dismantle systemic discrimination in access to formal employment and vocational training ( De Soto et al., 2005). Albania’s post-socialist economic transition embraced neoliberal growth without redistributive safeguards, exacerbating inequality along racialised and classed lines.

Housing conditions further reflect structural neglect. Many Roma and Egyptian families live in informal settlements lacking secure tenure, sanitation, or legal protection against eviction ( Open Society Foundation for Albania, 2014; UNDP, 2019). Housing strategies have largely focused on regularization or relocation, but implementation has been inconsistent, underfunded, and disconnected from community participation. The result is a continued pattern of displacement without redress, often under the guise of urban development ( United Nations Development Programme, 2019). Again, the consensus-oriented nature of these policies, designed to demonstrate compliance with international standards, tends to paper over deeper power inequalities and fails to involve affected communities in shaping outcomes.

While these inclusion strategies may appear progressive, they reflect a technocratic consensus model that seeks to avoid political confrontation, thereby reinforcing rather than resolving exclusion. The theoretical framework proposed in this article, drawing on agonistic pluralism and critical theories of the Capitalocene, argues that meaningful transformation requires engaging with conflict, not avoiding it. The failure of consensus-based reforms suggests that structural change emerges through struggle, contestation, and the politicization of inequality.

In contrast to the failure of consensus-oriented approaches, recent examples of agonistic interventions demonstrate the political agency of marginalized communities and the potential of conflict to disrupt exclusionary systems. These cases are analytically selected to illustrate how conflict functions as a mechanism linking structural inequality to transformative change.

A landmark instance is the European Court of Human Rights (ECHR) ruling on June 1, 2022, in favor of five Roma and Egyptian families who challenged the ethnic segregation of their children at the ‘Naim Frashëri’ elementary school in Korçë. With support from the European Roma Rights Centre, the families bypassed domestic legal systems and filed the case internationally, claiming a violation of Article 1, Protocol 12 of the European Convention on Human Rights. The ECHR concluded that Albania had failed to take effective desegregation measures and upheld systemic racial discrimination in education ( Gazeta Shqiptare, 2022).

This case exemplifies agonistic politics in Chantal Mouffe’s sense: political confrontation with state structures becomes a vehicle for democratic renewal. The success of the case lay not only in the ruling itself, whose long-term effects remain to be seen, but in the act of contestation, which disrupted Albania’s self-narrative of inclusive governance. It forced the state, and by extension, EU observers, to confront the gap between policy rhetoric and lived experience. In this sense, the case pushed for social change by shifting public discourse and legitimizing Roma and Egyptian claims in international law.

A second example of agonistic mobilisation occurred during the COVID-19 pandemic, when Roma and Egyptian communities organized protests against their exclusion from emergency food aid. While state narratives emphasized universal assistance, protestors revealed that many Roma and Egyptians were systematically left out due to a lack of documentation, discriminatory distribution practices, and bureaucratic inaccessibility ( Dervishi et al., 2022b). These actions, although small in scale, succeeded in drawing media attention and pressuring local governments to acknowledge their failure.

These examples, legal action and public protest, show that conflict is not a breakdown of democratic order but a sign of its vitality. Agonistic interventions challenge the myth of neutrality underpinning technocratic inclusion policies and expose the structural violence that such policies obscure. As Albania aspires to join the EU, these conflicts highlight the importance of institutionalizing dissent and recognizing that justice requires more than policy—it requires political confrontation and community-led resistance.

## 5. The role of conflict and suffering in transformation

Social transformation is often catalyzed by conflict, particularly when marginalized communities confront entrenched inequalities and systemic exclusions. Rather than being interpreted as a breakdown of order, conflict is increasingly understood in critical scholarship as a mechanism through which inequality becomes visible and politically contestable ( Fraser, 2018).

One such case is the 2018 evictions in Shkodra, where Roma families were forcibly removed from informal settlements. Local NGOs and community actors responded with a combination of protest and legal advocacy, resulting in temporary injunctions that suspended parts of the eviction process ( Open Society Foundation for Albania, 2014). Empirically, this case demonstrates how institutional recognition of claims can emerge only after public contestation, rather than through administrative procedures alone.

A similar pattern is visible during the COVID-19 pandemic, when Roma and Egyptian communities experienced disproportionate economic and food insecurity. A contemporaneous report documented severe deprivation, including food shortages among affected households ( Kryeziu, 2020). In response, protests in cities such as Fier and Elbasan emerged demanding access to emergency aid.

Importantly, these protests are treated as empirical evidence of political visibility rather than as symbolic acts. They illustrate how suffering becomes politically actionable only when it is publicly articulated, disrupting dominant narratives of universal crisis response.

Across these cases, a consistent empirical pattern emerges: marginalized groups often rely on intermediary actors—NGOs, activist networks, and community organisations—to translate lived suffering into institutional claims. After the 2018 floods, for example, NGOs worked alongside Roma representatives to coordinate emergency relief and educational continuity for displaced children ( European Commission, 2018; World Bank, 2019).

Empirically, this indicates that state responses tend to be reactive and short-term, while non-state actors often provide the continuity required for recovery and claim-making.

At the same time, the analysis does not assume that such interventions are sufficient for structural transformation. Rather, they highlight a key limitation observable across cases: while conflict can generate visibility and temporary redress, it does not automatically resolve underlying structural inequalities.

Finally, these empirical patterns intersect with environmental vulnerability. Roma communities are disproportionately affected by flooding and inadequate infrastructure due to their housing conditions. While this is consistent with broader environmental justice literature, in the Albanian case it remains largely absent from policy framing, suggesting a gap between environmental governance and lived exposure.

## 6. Critical analysis of just transition policies

Just transition has become a prominent policy frame in climate and development governance, yet its application often diverges significantly from the lived realities of marginalized groups ( Bueno Patin & Stapper, 2025). While policy discourse frequently emphasizes fairness and inclusivity, the empirical evidence from implementation contexts such as Albania suggests a persistent gap between normative commitments and actual outcomes.

In Albania, the National Action Plan for Roma and Egyptian Integration illustrates this tension. Although the plan includes formal objectives in employment, education, and health, available evaluations indicate limited and uneven implementation, particularly for Roma and Egyptian communities ( European Commission, 2018). This reflects a broader pattern in which inclusion is primarily addressed through administrative planning rather than structural transformation of inequality.

Empirical studies and institutional reports consistently highlight persistent disparities in education, employment, and housing. For example, Roma and Egyptian workers remain disproportionately concentrated in informal and precarious employment ( UNDP, 2019), while housing insecurity and informal settlements continue to affect many families ( UNDP, 2019). These outcomes indicate that policy instruments focused on “integration” have not substantially altered underlying structural conditions.

Disregarded social issues arise from focusing on Just Transitions infrastructures as their primary framework ( Butler et al., 2016). This gap remains at the core of Why Toils refuse to acknowledge alongside Schlosberg underlines this matter ( 2007): “the gap between idealized policy rhetoric and the lived realities of disadvantaged communities.” In nearly all cases, women, ethnic minorities or low-income earners advertising adequate social policies face multi-dimensional discrimination due to an insufficient green discourse providing merely timid digits. “Mainstream” does not approach these problems in any reliable manner.

Equally suppressive is trying to implement greener goals-planned obsolescence or change economic structure capitalism parallel within transition focused on socio-environmental growth ( Piggot *et al., *2019). For example, retraining programs for ex-coal miners often overlook the informal sectors where Roma, Egyptians, and other marginalized groups work ( Qejvanaj, 2021). Such policies are designed without considering the socio-economic and cultural realities of marginalized communities. These policies do prominent harm by deepening inequalities instead of addressing them.

Rather than treating these outcomes as isolated policy failures, this study interprets them as evidence of a broader governance approach in which inclusion is often framed in technical rather than political terms. In this framing, inequality is managed through policy delivery mechanisms, while the underlying power relations that produce exclusion remain largely unaddressed ( Ciplet & Harrison, 2020).

At the same time, the empirical material also shows that exclusion is not passively experienced. During the COVID-19 pandemic, for instance, Roma and Egyptian communities in several municipalities publicly protested exclusion from emergency assistance and food distribution ( Dervishi et al., 2022b). These events demonstrate that marginalized groups actively contest exclusion when institutional mechanisms fail to ensure equitable access. Similarly, cases of legal mobilization, including litigation before the European Court of Human Rights concerning school segregation, show how rights-based claims are used to challenge structural discrimination in education ( Gazeta Shqiptare, 2022).

Within this study, these events are not treated as isolated or anecdotal episodes but as analytically selected cases that illustrate broader patterns of conflict and exclusion within policy implementation. They show how gaps between policy design and lived experience can become sites of public contestation.

From a theoretical perspective, these findings suggest that consensus-oriented policy models tend to underplay conflict as a constitutive dimension of justice. However, it is important to emphasize that this interpretation is derived from a limited set of cases and secondary sources, and should therefore be read as indicative rather than exhaustive.

Overall, the evidence suggests that Just Transition policies in Albania remain largely aligned with technocratic and consensus-based governance approaches, which prioritize formal inclusion while often failing to address structural inequality in a substantive way. This highlights the need for policy frameworks that more explicitly recognize contestation and conflict as part of governance rather than as disruptions to it.

Fostering diverging opinions while accepting genuine conflicts that emerge when policies disregard the realities of life for marginalized groups engage is what defines agonistic approaches to Just Transition.

## 7. Pathways for justice-oriented transitions

Over time, justice has become a critical concern in the context of sustainability transitions, assessed both as an objective and as a framework within which to consider their governance and legitimacy. In the context of “Capitalocene,” which shifts attention from Anthropocene blame to capitalism’s violent systemic-ecological world regime, the demand for justice goes beyond distributional equity. It fundamentally requires transformation and reparative action along with thoroughly confronting inequality, exploitation, and environmental harm’s sociohistorical roots. For these reasons, justice-focused transitions must shift from mere technical fixes to comprehensive changes that dismantle the dominant systems of accumulation, extraction, and dispossession.

As such, the notion of Just Transition serves as an essential starting framework. Developed within labor activism to mitigate economic restructuring impacts on workers, Just Transition now also includes environmental ( Goodman, 2018) and climate justice ( Harper et al., 2009) along with intersectional race, gender, ( Keddie, 2012) and coloniality-based concerns ( Martínez-Alier, 2020). But more recently it has been highlighted (e.g., McCauley & Heffron, 2018) that Just Transition needs to be placed under other more encompassing theoretical frames that integrate restorative, procedural logics alongside recognition-based and epistemic forms of justice.

Instead of considering restorative justice as an addition or enhancement of Just Transition, we suggest viewing it as a profound complement that integrates both agonistic and deliberative approaches to democratic participation.

An effective transition grounded on justice principles requires simultaneously reallocating resources, recognizing past injustices, and restructuring engagement. The inclusion and empowerment of historically marginalized and disenfranchised groups as active agents of change is vital for this process. Policy-making informed by participatory recognition-based justice grants agency to those most affected by the climate and ecological crises dominantly shapes the transition.

Agyeman et al. (2012) stress that environmental justice must go beyond distribution to include participatory and procedural dimensions. Citizens’ assemblies are often celebrated for their deliberative inclusivity; however, these formats may inadvertently reproduce some inequalities they set out to dismantle. In contexts characterized by stark power disparities, the drive for consensus can become a means to exclude, silence dissenting voices or stall transformative efforts. A justice-oriented pathway should focus on balancing deliberation with more agonistic forms of politics that embrace contestation, resistance and conflict as essential elements of democracy.

Marginalized groups have historically relied on protest, lawsuits, and other forms of direct action to confront systemic injustice. These actions should be recognized as part the transition architecture framework, rather than being written off as fringe antagonistic interruptions. Such agonistic methods challenge the structural reasoning driving the Capitalocene and force transitions beyond pure technocratic concession.

In this regard, we advocate for a triadic framework of justice that consists of three interconnected aspects: first, inclusion-based policymaking that allows access and participation in all groups around the given structures to achieve distributive and procedural reforms; second, conflictual systems that allow constructional dispute disagreement space. These mechanisms recognize conflict and dissent as vital elements for democratic change; third, practices which strive to correct injury by accepting, repairing, and reconciling ‘doing wrong’ through intentional acts of undoing harm or injustice counteraction describes restorative injustices.

Zehr (2002) defines restorative justice as enabling a collective reckoning which permits communities to articulate harm, determine accountability, and collaboratively establish means of reparation. This is crucial in the context of climate injustice for Indigenous peoples and other ethnically marginalized groups who are descendants from long colonial resource-raping systems alongside epistemic erasure. Restorative frameworks can also fuel broader systemic change; Canada’s Truth and Reconciliation Commission is an example of this.

Crucially not engaging in conflict does not mean accepting defeat when practicing restorative justice.

Still, both can work together: While some practices disrupt dominant power configurations, other restorative approaches reweave fractured social relations and cultivate conditions for enduring reconciliation. Collectively, they expand the democratic horizons of transition processes and allow conflict as well as compassion, dissent alongside repair.

Thus, justice transitions need participation modes like activism, protest, deliberation and storytelling tooperate within a single system. According to Escobar (2016) and Mansbridge et al. (2012), democracy in complex systems requires polycentric formal and informal civic structures with systemic functions.

The situated nature of justice claims is illustrated further in case studies. In South Africa, Just Transition efforts within the coal sector have emphasized direct compensation along with oversight mechanisms which has stemmed from historically low trust in the state ( Wilgosh et al., 2022). On the other hand, African-led initiatives elsewhere on the continent are focused more on community-led deliberation alongside local knowledge ( Otlhogile & Shirley, 2023). These examples emphasize plural contextual frameworks that are responsive to culture history institutional specificity.

In the context of Albania, and more broadly in post-socialist transition settings, Just Transition policies are largely embedded within broader development and EU integration frameworks. While these frameworks introduce important social inclusion goals, their implementation often prioritizes institutional alignment and policy compliance over structural redistribution or meaningful participation of marginalized communities ( European Commission, 2018).

The empirical material discussed in this study suggests that effective justice-oriented transitions require moving beyond this narrow framing. First, there is a need to strengthen participatory mechanisms so that affected communities are not only beneficiaries of policy but also active participants in its design and evaluation. Existing evidence from community responses, including protest and legal mobilization, indicates that marginalized groups already seek to influence policy outcomes when formal channels are insufficient.

Second, the analysis suggests that policy frameworks should more explicitly recognize conflict as a legitimate component of democratic governance. Rather than treating contestation as a failure of consensus, conflict can be understood as an indicator of unresolved inequality that requires institutional attention.

Third, while restorative and recognition-based approaches have been widely discussed in the literature, their relevance in this context lies in their potential to complement rather than replace redistributive policy. However, the effectiveness of such approaches remains dependent on institutional capacity and political will, which varies significantly across contexts.

It is important to emphasize that these implications are drawn from a qualitative and interpretive analysis of selected cases and secondary sources. As such, they should not be understood as universal policy prescriptions but as analytically grounded insights that may inform future comparative and empirical research.

## 8. Conclusion

This article contributes to the growing body of scholarship on justice-oriented transitions by emphasizing the transformative potential of conflict, particularly for marginalized communities. By focusing on the experiences of Albania’s Roma and Egyptian populations, it illustrates how conflict-driven change can illuminate the deep inequalities embedded in climate justice discourses and practices.

The findings underscore that many current Just Transition policies, despite their rhetorical commitment to equity, often fail to meaningfully engage marginalized groups. This is especially evident in cases such as Albania’s National Action Plan for Roma and Egyptians, where well-intentioned policy frameworks have not translated into inclusive outcomes ( European Commission, 2018). The disconnect between policy ambition and lived reality highlights the insufficiency of transition processes that prioritize technocratic consensus over political contestation.

A key insight of this analysis is the inadequacy of consensus-based approaches that dominate mainstream Just Transition discourse. Such approaches often depoliticize transition by seeking to neutralize dissent and obscure structural inequalities. Drawing on Mouffe’s theory of agonistic pluralism, this article argues for the necessity of institutionalizing dissent rather than suppressing it. Agonistic approaches recognize conflict as a generative force for democratic renewal and justice. In this context, dissent becomes a mechanism for marginalized communities to assert political agency and demand structural change.

This perspective aligns with Swyngedouw’s (2011) critique of post-democratic governance, which cautions against the foreclosure of egalitarian political spaces under the guise of technocratic management. Transition governance that sidelines dissent reproduces the same capitalist and colonial logics that underpin the Capitalocene. Within this frame, the experiences of Roma and Egyptian communities in Albania reflect broader dynamics in which climate and sustainability transitions risk entrenching the hegemonic systems of racialized and class-based exclusion.

From a conflict theory standpoint, the struggles of these communities highlight the need to politicize Just Transition processes by foregrounding issues of power, historical harm and redistribution. Pellow (2023) argues that conflict recognition is essential to address past injustices and for preventing their reproduction.

The study therefore argues—at an interpretive level—that justice-oriented transitions may benefit from approaches that recognise conflict as part of democratic governance rather than as a disruption to it. This argument is supported by the selected empirical material but remains analytical rather than predictive in scope.

At the same time, the analysis points to important limitations. The study is based on qualitative document analysis and selected case evidence, rather than primary data or large-scale comparative datasets. As a result, the findings are not intended to be generalisable, but instead to offer a theoretically informed interpretation of documented patterns of exclusion and contestation.

Future research could strengthen and extend these findings through comparative empirical studies across different national contexts, longitudinal analysis of policy implementation outcomes, and primary data collection involving affected communities. Such work would allow for a more systematic evaluation of how conflict, governance, and justice interact in practice.

In conclusion, while the evidence presented here is limited in scope, it suggests that transition processes which rely primarily on consensus-based governance risk underestimating the role of political conflict in shaping outcomes. Drawing on conflict theory, agonistic pluralism, and Capitalocene critique, the article advances the theoretical proposition that dissent is not external to justice-oriented transitions but may be constitutive of them. However, this proposition should be read as an interpretive contribution grounded in selected empirical cases, rather than as a definitive empirical generalisation.

## Ethics and consent

Ethical approval and consent were not required.

The research is based exclusively on qualitative policy analysis and the review of publicly available documents and academic literature. It did not involve human participants, personal data, or primary data collection.

## Data Availability

This study is based on qualitative policy analysis and a critical review of publicly available policy documents and academic literature. No primary empirical data were generated or analysed during this study. All documents cited are publicly accessible through official institutional websites and the sources listed in the reference section. As the article does not involve original quantitative or qualitative datasets, there are no underlying data (e.g., raw datasets, numerical values, or image data) to deposit in a public repository.
