# Supplementary material for: From marginalization to transformation: Examining structural injustices and policy impacts on Albania’s Roma and Egyptian communities
Source: Open Res Eur. 2026 Jul 11;6:69. Originally published 2026 Mar 10. [Version 2] doi: 10.12688/openreseurope.23119.2 (PMC13555071; doi:10.12688/openreseurope.23119.2)
Supplement: Peer review history

# Reviewer report: Reviewer response for version 2

**DOI:** 10.21956/openreseurope.25775.r77452

**Published:** 2026-07-27

**Authors:** Nilam Firmandayu

**Affiliations:** 1 Faculty of Law, Universitas Gadjah Mada, Yogyakarta, Indonesia

Reviewer Comments

1. In the Introduction, consider adding a definition or conceptual framework of “just transition” to clarify the parameters of the transition the article seeks to achieve, particularly in the context of reformulating the issues of social and environmental justice. While the article currently provides a COVID-19 case illustration, it should also incorporate an environmental example, particularly one affecting Albania’s Roma and Egyptian communities. This would reduce abrupt shifts in the discussion and ensure consistency with the environmental scope of the article.
2. In the fifth paragraph of the Introduction, briefly introduce the theoretical framework to demonstrate that the identified issues should be addressed through a coherent theoretical approach rather than on a case-by-case basis.
3. In Section 5, "The Role of Conflict and Suffering in Transformation," you may consider, if appropriate, incorporating the concept of “legal mobilization” to complement the analysis. Within legal scholarship, the activities of local NGOs and community actors are often understood through the framework of “legal mobilization”, whereby marginalized groups pursue their rights through legal instruments, such as initiating litigation or seeking judicial review. Consistent with your argument, legal mobilization conceptualizes collective conflict as a means of fostering transformation, while emphasizing the more substantive role of legal mechanisms in advancing social change.
4. In Section 6, "Critical Analysis of Just Transition Policies," I noted that the discussion does not yet address specific environmental issues affecting Albania’s Roma and Egyptian communities. You may consider incorporating relevant examples to strengthen the analysis and ensure its alignment with the article’s environmental focus.
5. In Section 7, "Pathways for Justice-Oriented Transitions," I consider this to be the central discussion of your article. Within the proposed triadic framework of justice and effective justice-oriented transitions, the first component concerning group access and participation in policymaking could be strengthened by incorporating distributive and procedural reform parameters to better operationalize the claim of "meaningful" participation presented in the Abstract and Introduction. For example, participation should extend from policy planning and deliberation to decision-making. However, this alone is insufficient to ensure meaningful participation if policymakers fail to “accommodate” public concerns and objections in the policy revision process.
6. The discussion on conflict as a driver of transformation would be more compelling if it also addressed the structural disadvantages faced by marginalized groups, which often result in unfavorable outcomes in judicial proceedings or law enforcement processes due to power imbalances, social status, collusion, and discrimination. The analysis could then further explore what an ideal justice framework should entail in addressing these barriers, together with concrete strategies for ensuring meaningful participation and effective access to justice for marginalized communities.
7. In the Conclusion, there is no need to restate the study's methodology, particularly in paragraphs 7 and 8. Instead, the conclusion would be strengthened by presenting the principal findings more specifically, particularly the proposed triadic framework of justice and effective justice-oriented transitions, as elaborated through the perspectives of conflict theory, agonistic theory, and the Capitalocene.
8. Writing structure: It would be preferable for each paragraph to contain at least three sentences. Consider combining shorter paragraphs into a single, more cohesive paragraph where appropriate, particularly in the Method section and elsewhere throughout the manuscript.

Is the case presented with sufficient detail to be useful for teaching or other practitioners?

Yes

Is the work clearly and accurately presented and does it cite the current literature?

Yes

If applicable, is the statistical analysis and its interpretation appropriate?

Not applicable

Are all the source data underlying the results available to ensure full reproducibility?

Yes

Are the conclusions drawn adequately supported by the results?

Partly

Is the background of the case’s history and progression described in sufficient detail?

Partly

Reviewer Expertise:

Law, Legal Theory, Environmental Law, Administrative Law, Constitutional Law, Public Policy

I confirm that I have read this submission and believe that I have an appropriate level of expertise to confirm that it is of an acceptable scientific standard, however I have significant reservations, as outlined above.

# Reviewer report: Reviewer response for version 2

**DOI:** 10.21956/openreseurope.25775.r77238

**Published:** 2026-07-22

**Authors:** Phoebe Koundouri

**Affiliations:** 1 Earth Sciences, University of Cambridge (Ringgold ID: 2152), Cambridge, England, UK; 2 Department of International and European Economic Studies, Athens University of Economics and Business, Athens, Attica, Greece

I thank the author for carefully addressing my previous comments. The revised manuscript has improved substantially and is now considerably stronger in terms of methodological transparency, theoretical coherence, and overall clarity.

 The addition of a dedicated methodology section, including the source selection strategy, analytical approach, and study limitations, greatly enhances the transparency and rigor of the paper. The historical analysis is now more structured and provides clearer links between historical marginalization and present-day inequalities. The integration of conflict theory, agonistic pluralism, and the Capitalocene has also been significantly strengthened, resulting in a more coherent conceptual framework. 

 The author has also addressed my concerns regarding the distinction between empirical evidence and theoretical interpretation by explicitly acknowledging the illustrative nature of the selected cases and the limitations of the study. Although the manuscript remains primarily conceptual, this is now appropriately reflected in the discussion and conclusions.  

 Overall, I believe the author has adequately addressed my previous comments. The remaining issues are minor and do not affect the overall quality of the manuscript. I therefore recommend **approval** of the revised version.

Is the case presented with sufficient detail to be useful for teaching or other practitioners?

Partly

Is the work clearly and accurately presented and does it cite the current literature?

Partly

If applicable, is the statistical analysis and its interpretation appropriate?

Not applicable

Are all the source data underlying the results available to ensure full reproducibility?

Partly

Are the conclusions drawn adequately supported by the results?

Partly

Is the background of the case’s history and progression described in sufficient detail?

Partly

Reviewer Expertise:

Environmental and Resource Economics; Sustainable Development; Climate Change Economics; Integrated Assessment Modelling; Climate Policy and Governance; Just Transition and Climate Justice; Natural Resource Management; Interdisciplinary Sustainability Science.

I confirm that I have read this submission and believe that I have an appropriate level of expertise to confirm that it is of an acceptable scientific standard.

# Reviewer report: Reviewer response for version 1

**DOI:** 10.21956/openreseurope.25011.r71307

**Published:** 2026-04-22

**Authors:** Vikniswari Vija Kumaran

**Affiliations:** 1 Universiti Sains Malaysia, Gelugor, Penang, Malaysia

The manuscript makes a valuable and timely conceptual contribution by examining the role of conflict, suffering, and structural injustices in driving transformative processes within Albania’s Roma and Egyptian communities based on a qualitative analysis of policy documents and academic literature. 

Comments:

 1. The core argument is that conflict should be understood as a productive and necessary force in achieving justice-oriented transformation, rather than something to be minimized in governance and policy frameworks.

 2. The manuscript addresses an important and underexplored topic. However, it reads more as a theoretical essay with illustrative cases than as a fully developed methodologically rigorous case study.

 3. The study lacks a clearly defined methodology. While it states that it is based on policy analysis and literature review, there is:

 i. No explanation of how sources were selected

 ii. No inclusion/exclusion criteria

 iii. No analytical framework or method (e.g., thematic analysis)

 iv. No clear scope or boundaries of the dataset

 4. The empirical component is limited in terms of:

 i. Evidence is illustrative rather than analytical

 ii. Systematic case study approach

 iii. There is little use of empirical indicators (e.g., statistics on education, employment, housing)

 5. The theoretical discussion is strong but overly dense and sometimes repetitive and the empirical analysis is comparatively weak. The paper leans heavily toward a theoretical essay. 

 6. Overall, the selection and interpretation of sources are not transparent and the analytical process is not documented. 

 7. The manuscript needs a limitations section addressing the scope of evidence and generalizability. 

 8. The manuscript lacks clear policy recommendations and practical implications.

Is the case presented with sufficient detail to be useful for teaching or other practitioners?

Partly

Is the work clearly and accurately presented and does it cite the current literature?

Partly

If applicable, is the statistical analysis and its interpretation appropriate?

Not applicable

Are all the source data underlying the results available to ensure full reproducibility?

Partly

Are the conclusions drawn adequately supported by the results?

Partly

Is the background of the case’s history and progression described in sufficient detail?

Partly

Reviewer Expertise:

Economics

I confirm that I have read this submission and believe that I have an appropriate level of expertise to confirm that it is of an acceptable scientific standard, however I have significant reservations, as outlined above.

# Response

**Published:** 2026-04-26

**Authors:** Eglantina Dervishi

**Affiliations:** Pedagogy and Psychology, University of Tirana, Albania, Tirana, Albania, Albania

**From marginalization to transformation: Examining structural injustices and policy impacts on Albania’s Roma and Egyptian communities** **Platform: Open Research Europe (Open Access, European Commission)**

   Dear Editor, Thank you for the opportunity to revise our manuscript. We also thank the Reviewers for their helpful feedback and suggestions. Please find below our point-by-point reply (in italics) to the concerns they raised. We hope that you find that our revision satisfactorily addresses all the critical points that have been highlighted. All changes in the manuscript have been made according to the suggestions pointed out by the reviewers. Sincerely, **Response to Reviewer 1 Comments** We sincerely thank Reviewer 1 for the careful and constructive evaluation of our manuscript. We appreciate the recognition of its conceptual contribution and relevance to debates on justice-oriented transitions, structural inequality, and marginalized communities in Albania. The feedback has been extremely helpful in improving methodological clarity, empirical grounding, theoretical balance, and overall presentation. Below, we respond to each comment in detail. **1. Conceptual Contribution and Core Argument** ***Reviewer comment:*** *The manuscript makes a valuable conceptual contribution by reframing conflict as a productive force in justice-oriented transformation* **.** **Response:**

 We thank the reviewer for this positive assessment. In the revised manuscript, we further clarify and strengthen the central argument that conflict should not be understood as a phenomenon to be minimized, but as a constitutive and productive force in processes of justice-oriented transformation. We have refined the theoretical framing to better integrate agonistic pluralism, conflict theory, and political ecology perspectives, explicitly linking these to the lived experiences of Roma and Egyptian communities in Albania. **2. Historical Background and Analytical Structure** ***Reviewer comment:*** *The historical narrative is broad and descriptive, lacking analytical structure and clearer causal links.* **Response:**

 We agree with this important observation and have substantially revised the historical section. The revised manuscript now:

- Organizes the historical analysis into a clearer chronological sequence (Ottoman → socialist → post-1990 transition)
- Strengthens causal linkages between historical exclusion and present-day inequalities
- Integrates empirical indicators from UNDP and European Commission reports
- Frames history explicitly as a **path-dependent process of structural inequality formation**, rather than a descriptive background

We also acknowledge that the analytical depth of historical interpretation is influenced by the disciplinary lens as an author (psychology). We prioritize lived experience and structural perception over formal historiographic reconstruction. **3. Theoretical Density and Clarity** ***Reviewer comment:*** *The theoretical section is strong but dense, sometimes repetitive, and affects clarity.* **Response:**

 We have revised the theoretical section to improve clarity and readability. Specifically:

- Reduced repetition across theoretical frameworks
- Clarified the distinct analytical role of conflict theory, agonistic pluralism, and Capitalocene
- Strengthened integration between theory and empirical cases

The revised manuscript now uses theory more explicitly as an analytical lens applied to empirical material rather than as a standalone theoretical discussion. **4. Empirical Grounding and Balance** ***Reviewer comment:*** *The empirical dimension is limited, and some claims appear normative rather than evidence-based.* **Response:**

 We acknowledge this limitation and have strengthened the empirical grounding by:

- Incorporating relevant indicators (education, employment, housing) from international datasets
- Strengthening contextualization of case examples (ECHR ruling, COVID-19 protests, evictions)

We also explicitly acknowledge that the limited empirical base is partly due to the **scarcity of systematically documented data on Roma and Egyptian communities**, reflecting broader structural invisibility in official and academic sources. **5. Methodological Transparency** ***Reviewer comment:*** *The study lacks a clearly defined methodological framework.* **Response:**

 We fully agree and have substantially strengthened the methodology section. The revised manuscript now specifies:

- Qualitative interpretive research design
- Purposive sampling of policy and academic sources
- Inclusion/exclusion criteria
- Thematic and critical discourse analysis
- Scope and limitations of secondary data

We also explicitly clarify that no primary data were collected and that the analysis is interpretive in nature. **6. Conclusions and Evidence Alignment** ***Reviewer comment:*** *Conclusions are broadly consistent but sometimes extend beyond evidence.* **Response:**

 We have revised the conclusions to ensure stronger alignment with empirical material. Specifically:

- Reduced overgeneralization
- Clearly distinguished empirical findings from theoretical interpretation
- Added a limitations section

  We thank Reviewer 1 for recognizing the manuscript’s relevance and contribution.

# Reviewer report: Reviewer response for version 1

**DOI:** 10.21956/openreseurope.25011.r71304

**Published:** 2026-04-02

**Authors:** Phoebe Koundouri

**Affiliations:** 1 Earth Sciences, University of Cambridge (Ringgold ID: 2152), Cambridge, England, UK; 2 Department of International and European Economic Studies, Athens University of Economics and Business, Athens, Attica, Greece

This manuscript explores the role of conflict, suffering, and structural inequality in shaping transformation processes among Albania’s Roma and Egyptian communities. It proposes a conceptual framework that brings together conflict theory, agonistic pluralism, and the Capitalocene, and applies this lens to a qualitative case study based on policy analysis and existing literature. The core argument is that dominant consensus-driven “Just Transition” approaches are insufficient to address systemic inequalities and that conflict should instead be understood as a necessary and productive force in achieving justice-oriented transformation. The paper illustrates this argument through selected examples, including legal contestation and grassroots mobilization.

 The article addresses a highly relevant and underexplored topic at the intersection of climate justice, governance, and social exclusion. It offers an original conceptual contribution, although its empirical grounding and methodological clarity remain limited.

 The manuscript provides a useful general overview of the historical marginalization of Roma and Egyptian communities, referring to the Ottoman period, the socialist era, and the post-1990 transition. It also links these developments to contemporary policy frameworks and inclusion strategies. However, the historical narrative remains relatively broad and descriptive, without sufficient depth or systematic structure. The progression of events is not presented in a clearly articulated analytical sequence, and the causal connections between historical exclusion and present-day inequalities could be more explicitly developed. Greater use of empirical indicators and a clearer chronological organization would significantly strengthen this section and enhance the analytical clarity of the case study.

 The manuscript demonstrates a strong engagement with interdisciplinary literature, particularly within critical theory, environmental justice, and sustainability transitions. The integration of conflict theory, agonistic pluralism, and the Capitalocene is conceptually rich and represents a notable strength of the paper. However, the presentation is at times overly dense, and the theoretical discussion occasionally becomes repetitive, which affects clarity. Moreover, while the theoretical grounding is robust, the empirical dimension is less developed, and some claims appear more normative than evidence-based. The manuscript would benefit from a clearer balance between theory and empirical material, as well as a stronger engagement with recent, context-specific studies on Albania or comparable regional cases.

 The author clearly states that the study relies exclusively on publicly available policy documents and academic literature, and that no primary data were generated. While this is appropriate for the type of study conducted, the manuscript lacks sufficient methodological transparency to ensure reproducibility. In particular, there is no detailed explanation of how sources were selected, what criteria guided their inclusion, or how they were analyzed. The absence of a clearly defined methodological protocol limits the transparency and rigor of the analysis. Providing a more explicit description of the research design, including the analytical approach and the scope of the reviewed material, would significantly improve the scientific robustness of the study.

 The conclusions are broadly consistent with the theoretical framework and are supported by illustrative examples presented in the text, such as the European Court of Human Rights ruling and the COVID-19-related protests. However, the empirical basis of the analysis remains relatively limited and selective. The examples function more as illustrative cases rather than as part of a systematic analysis, and in some instances the conclusions extend beyond what is directly supported by the evidence provided. The manuscript would benefit from a clearer distinction between empirical findings and theoretical interpretation, as well as from a more explicit acknowledgment of the scope and limitations of the case study.

 The article has strong potential for teaching, particularly in courses related to environmental justice, sustainability transitions, and critical policy studies. Its conceptual contribution is valuable and thought-provoking. However, its usefulness for practitioners is currently limited by the highly theoretical nature of the presentation and the absence of clearly articulated policy implications. The manuscript would benefit from a more explicit discussion of how its findings can inform policy design and implementation, as well as from a more accessible presentation of key insights.

 This manuscript makes a timely and potentially important contribution to the literature on justice-oriented transitions by foregrounding the role of conflict and marginalization. Its conceptual ambition and interdisciplinary scope are commendable, and the focus on Roma and Egyptian communities in Albania addresses a significant gap in the literature.

 At the same time, the paper currently reads more as a theoretically driven essay than as a fully developed case study. The main limitations relate to the lack of methodological transparency, the relatively limited and unsystematic use of empirical evidence, and the need for greater clarity in linking theoretical arguments to the case analysis. Addressing these issues would substantially strengthen the manuscript and enhance its contribution to both academic and policy debates.

Is the case presented with sufficient detail to be useful for teaching or other practitioners?

Partly

Is the work clearly and accurately presented and does it cite the current literature?

Partly

If applicable, is the statistical analysis and its interpretation appropriate?

Not applicable

Are all the source data underlying the results available to ensure full reproducibility?

Partly

Are the conclusions drawn adequately supported by the results?

Partly

Is the background of the case’s history and progression described in sufficient detail?

Partly

Reviewer Expertise:

Environmental and Resource Economics; Sustainable Development; Climate Change Economics; Integrated Assessment Modelling; Climate Policy and Governance; Just Transition and Climate Justice; Natural Resource Management; Interdisciplinary Sustainability Science.

I confirm that I have read this submission and believe that I have an appropriate level of expertise to confirm that it is of an acceptable scientific standard, however I have significant reservations, as outlined above.

# Response

**Published:** 2026-04-26

**Authors:** Eglantina Dervishi

**Affiliations:** Pedagogy and Psychology, University of Tirana, Albania, Tirana, Albania, Albania

**From marginalization to transformation: Examining structural injustices and policy impacts on Albania’s Roma and Egyptian communities** **Platform: Open Research Europe (Open Access, European Commission)**

   Dear Editor, Thank you for the opportunity to revise our manuscript. We also thank the Reviewers for their helpful feedback and suggestions. Please find below our point-by-point reply (in italics) to the concerns they raised. We hope that you find that our revision satisfactorily addresses all the critical points that have been highlighted. All changes in the manuscript have been made according to the suggestions pointed out by the reviewers. Sincerely, **Response to Reviewer 2 Comments** We sincerely thank Reviewer 2 for the detailed and constructive feedback. The comments have been extremely valuable in improving methodological transparency, empirical rigor, theoretical clarity, and policy relevance. Below we respond to each point. **1. Core Argument and Conceptual Framing** ***Reviewer comment:*** *Conflict is framed as a productive force rather than something to be minimized.* **Response:**

 We thank the reviewer for acknowledging the conceptual framing. The revised manuscript further clarifies that conflict is conceptualized as a structural and productive element of justice-oriented transformation, particularly in contexts of persistent exclusion and inequality. **2. Nature of the Study (Theoretical vs Case Study)** ***Reviewer comment:*** *The manuscript reads more like a theoretical essay than a rigorous case study.* **Response:**

 We agree and have clarified the epistemological positioning of the study. The manuscript is now explicitly framed as a **qualitative interpretive case study with strong theoretical grounding**, rather than a purely empirical case study. We emphasize interpretive analysis rather than statistical generalization. **3. Methodological Clarity** ***Reviewer comment:*** *No clear explanation of source selection, inclusion/exclusion criteria, analytical framework, or dataset scope.* **Response:**

 We have significantly expanded the methodology section, now including:

- Source selection strategy (purposive sampling)
- Inclusion/exclusion criteria
- Analytical method (thematic + discourse analysis)

**4. Empirical Limitations** ***Reviewer comment:*** *Evidence is illustrative rather than systematic; lack of indicators.* **Response:**

 We acknowledge this limitation and have strengthened empirical grounding by:

- Incorporating indicators from UNDP and European Commission reports
- Reframing case studies as analytically selected rather than exhaustive

We also explicitly note that limited empirical availability reflects structural under-documentation of marginalized communities, which is itself part of the inequality being analyzed. **5. Theoretical Density and Empirical Balance** ***Reviewer comment:*** *The theoretical discussion is strong but dense and repetitive, with weak empirical balance.* **Response:**

 We have streamlined the theoretical section by:

- Removing repetition across frameworks
- Clarifying distinct roles of each theory
- Improving readability

**6. Transparency of Source Selection** ***Reviewer comment:*** *The selection and interpretation of sources are not transparent.* **Response:**

 This is addressed through the expanded methodology section, which now clearly documents:

- Source identification and selection procedures
- Analytical approach (thematic and discourse analysis)
- Inclusion/exclusion criteria
- Scope limitations

**7. Limitations Section** ***Reviewer comment:*** *The manuscript lacks a limitations section.* **Response:**

 A dedicated limitations section has been added. It explicitly addresses:

- Qualitative and interpretive nature of the study
- Reliance on secondary data
- Non-exhaustive case selection
- Limited availability of structured data
- Restricted generalizability

We also explicitly acknowledge positional limitations of the analysis. **8. Positionality and Disciplinary Perspective (New Addition)** We now explicitly acknowledge that the analysis is influenced by our author’s **background in psychology**, which shapes the interpretive focus on:

- lived experience of suffering and exclusion
- perception of structural inequality
- psychosocial dimensions of conflict

This perspective necessarily limits historical-structural depth in favor of interpretive and experiential analysis. **9. Policy Implications** ***Reviewer comment:*** *Lack of policy implications and practical relevance.* **Response:**

 We have added a dedicated policy implications section outlining how findings can inform: We thank the reviewer for highlighting the need for clearer policy implications and practical relevance. In response, we have substantially revised the manuscript (particularly Sections 6 and 7 and the conclusion) to explicitly derive policy-relevant insights from the empirical material and strengthen their grounding in documented cases and policy analysis. The revised paper now shows how existing Just Transition and inclusion frameworks in Albania remain largely technocratic and consensus-driven, resulting in persistent inequalities in education, employment, and housing for Roma and Egyptian communities despite formal policy commitments. Building on empirical cases such as the ECHR ruling on school segregation and COVID-19-related exclusion from emergency support, we now explicitly articulate policy implications, including the need to shift from top-down inclusion models toward participatory governance, to recognize conflict and contestation as legitimate components of democratic policy processes, and to integrate redistribution, recognition, and meaningful participation in transition frameworks. Importantly, we clarify that these implications are interpretive and context-specific, given the study’s reliance on secondary data and selected case evidence, and are not intended as universal policy prescriptions. We also explicitly acknowledge the limitations of available documented data on these marginalized communities and note that the author’s psychological background informs a focus on lived experience and micro-level impacts, while constraining macro-level policy generalization. We believe these revisions significantly enhance the manuscript’s policy relevance while maintaining its theoretical and interpretive scope. We thank Reviewer 2 for the constructive feedback.
